# How to Use and Report on *p*-values

**DOI:** 10.5334/pme.1324

**Published:** 2024-04-26

**Authors:** Christy K. Boscardin, Justin L. Sewell, Martin G. Tolsgaard, Martin V. Pusic

**Affiliations:** 1 Department of Medicine, University of California, San Francisco, California, US; 2 Department of Anesthesia, University of California, San Francisco, California, US; 3 Medical Education, Copenhagen Academy for Medical Education and Simulation, Copenhagen, DK; 4 Harvard Medical School, Boston, MA, US

## Abstract

The use of the p-value in quantitative research, particularly its threshold of “P < 0.05” for determining “statistical significance,” has long been a cornerstone of statistical analysis in research. However, this standard has been increasingly scrutinized for its potential to mislead findings, especially when the practical significance, the number of comparisons, or the suitability of statistical tests are not properly considered. In response to controversy around use of p-values, the American Statistical Association published a statement in 2016 that challenged the research community to abandon the term “statistically significant”. This stance has been echoed by leading scientific journals to urge a significant reduction or complete elimination in the reliance on p-values when reporting results. To provide guidance to researchers in health professions education, this paper provides a succinct overview of the ongoing debate regarding the use of p-values and the definition of p-values. It reflects on the controversy by highlighting the common pitfalls associated with p-value interpretation and usage, such as misinterpretation, overemphasis, and false dichotomization between “significant” and “non-significant” results. This paper also outlines specific recommendations for the effective use of p-values in statistical reporting including the importance of reporting effect sizes, confidence intervals, the null hypothesis, and conducting sensitivity analyses for appropriate interpretation. These considerations aim to guide researchers toward a more nuanced and informative use of p-values.

## Background

“*P* < 0.05” has long been afforded nearly unimpeachable status in the discourse of quantitative research and statistics, leading many researchers to confidently claim “statistical significance” for statistical comparisons regardless of practical significance, number of comparisons made, or appropriateness of statistical tests used. In response, the American Statistical Association (ASA) released a statement about *p*-values in 2016 including the declaration that “…*it is time to stop using the term ‘statistically significant’ entirely. Nor should variants such as ‘significantly different,’ ‘p < 0.05,’ and ‘non-significant’ survive, whether expressed in words, by asterisks in a table, or in some other way*” [1]. This statement led many prominent scientific journals, including *New England Journal of Medicine* (NEJM) and *Nature*, to either support significantly reducing or completely abandoning the use of *p*-values [2]. Since reporting *p*-values has been ubiquitous and considered a crucial part of the standard reporting of quantitative research for much of the last century and has provided a single and universally understood measure of “statistical significance”, the ASA statement ignited debate around the use of *p*-values, and dilemma among many research communities, including medical education. In this Statistical Points and Pitfalls, we reflect on this controversy and offer guidance on how researchers may best utilize (or not) *p*-values when reporting on medical education research and scholarship.

## What is a *p*-value?

The concepts of hypothesis testing and *p*-values are attributed to the seminal paper published by Karl Pearson in 1900 and popularized by Ronald Fisher in the 1920s [3,4]. Originating from hypothesis testing, **the *p*-value is defined as the probability of obtaining data equal to or more extreme than the data (results) observed, given that the null hypothesis is true**. The null hypothesis test for examining comparisons between groups can be stated as “null *Ho*: the unit describing the difference between groups = 0” (i.e., there is no difference between the groups). As an illustrative hypothetical example, imagine a study that examined the effect of novel simulation training on learner performance in the placement of a peripheral venous line. In this study comparing novel simulation training vs. “standard” training, the difference in the outcome was 5 points in favor of the novel simulation training group on a 100-point scale. A two-sample t-test was used to compare the mean difference between the two groups and obtained *p* = .04. In this case, *p* = 0.04 means there is only 4% probability of observing this 5-point difference (in either direction) between the two groups if in fact there was no difference between the groups (i.e., the null hypothesis that the difference is zero). In other words, there is only 4% probability that the differences found between the two groups were just due to chance (i.e., they are not “true differences”).

So then, where did the *p*-value < .05 threshold as the gold standard come from? The threshold for 0.05 is rather arbitrary and Fisher’s writing indicated he never intended it to be the single standard. As stated by Fisher in 1926, “…*If one in twenty does not seem high enough odds, we may, if we prefer it, draw the line at one in fifty or one in a hundred. Personally, the writer prefers to set a low standard of significance at the 5 percent point, and ignore entirely all results which fail to reach this level*” [5]. Despite the arbitrariness and the acknowledgment of the limitations of dichotomization of *p*-values for determining statistical significance, the threshold of <.05 gained popularity due to its simplicity and ease of interpretation.

## What are the common pitfalls when using *p*-value?

### Misinterpreting *p*-values for magnitude of difference

Since the *p*-value represents the probability (degree of likelihood), lower probability would signal that the null hypothesis (e.g, there is no difference between the group) may be unlikely. However, the *p*-value provides only indirect evidence to support or refute the null hypothesis. Researchers sometimes interpret the magnitude of a *p*-value as a measure of the effect size (i.e., the size of the difference found) or the strength of evidence for or against the null hypothesis. However, “more significant” *p*-values (e.g., *p* < .01 or *p* < .001) do not imply larger effect sizes. In fact, *p*-values alone do not provide any information about the magnitude or practical significance of an effect; other measures are needed to provide this information.

### Overemphasis on statistical significance

Researchers and reviewers often put excessive emphasis on statistical significance (*p* < 0.05) in educational research, yet statistical significance does not necessarily equate to practical or educational significance. Even if a study finds a statistically significant result, it is essential to consider other important factors like effect size (as described above), sample size (whether the study is insufficiently powered to a difference or is “overpowered” and will detect even a tiny numerical difference as statistically significant), and contextual factors unique to the study to determine the practical importance of a finding. Considering the example above, it would be appropriate to ask, “Is a 5-point difference on a 100-point scale educationally meaningful?”

### Under-emphasis on *p*-values greater than .05

A statistical result that does not meet the typical statistical significance threshold does not necessarily mean there is no effect present. **There is nothing magical about a *p*-value < 0.05**. The sample size, study design, sensitivity of the measurement to detect differences, and variability of the data, among other things, can all influence the researcher’s ability to detect an effect. Therefore, it is important to consider the entire body of evidence, including nonsignificant findings, when drawing conclusions from educational research.

### Dichotomization of *p*-values

Many researchers report the results of the study by stating whether the results are significant or not significant. As stated above, any *p*-value threshold that one sets is arbitrary, so dichotomizing the statistical significance as significant or not significant based on the 0.05 threshold (or any other threshold, for that matter) should be made with caution. As illustrated by McShane and Gal, previous studies have shown that emphasis on strict dichotomization, which is the current convention in many fields, leads to misinterpretation and hinders more integrative approaches to the interpretation of evidence [6].

## What are the key recommendations and points when using *p*-values?

### Report effect sizes and confidence intervals

Rather than reporting just the *p*-values, it is important to report effect sizes (e.g., Cohen’s *d* for mean differences, *R*^2^ for regression, or odds ratios for risk differences) and their associated confidence intervals (typically 95% CI). The magnitude of difference in the outcomes is often determined by effect sizes. Effect sizes provide a measure of the magnitude of an effect, while confidence intervals provide the precision (level of certainty) around the point estimate of the observed differences or the relationships. Sullivan and Feinn have provide a detailed description and guidelines around how to calculate and report on effect sizes [8].

The 95% confidence interval (CI) of the point estimate describes the precision and range of the potential difference between the two comparison groups. In the example above where a difference of 5 points between the two groups resulted in a p-value of 0.04, the 95% CI for the difference was (1, 9). This means that we are 95% confident that the true difference lies between 1 and 9 points, greatly expanding our understanding of “*p* = 0.04” [7]. While the 95% confidence interval reveals precision and range, the effect size indicates practical implications. The wider the 95% CI, the lower the precision in the estimates and the less reliable the interpretation of the findings (due to the higher level of uncertainty). It is also helpful to report the practical or educational significance of the findings (e.g., how would a 5-point difference in a 100-point assessment translate into differences in practice?). Report the results in the context of the research question and existing literature to guide the interpretation of the findings.

### Report the null hypothesis

Clearly define what the *p*-value represents and include the null hypothesis when reporting it. For example, state whether the *p*-value represents a one-tailed or two-tailed test, and how it relates to the null hypothesis. For the hypothetical study above, we could write: “We tested the null hypothesis of no difference in performance between simulation and standard training groups, using a two-tailed t-test in which either group could perform better or worse.”

### Interpret the *p*-values in the context of the sample size

As mentioned briefly above, a large sample size can lead to statistically significant *p*-values even for very small effects, while a small sample size may fail to reach a statistically significant *p*-value, even for very large effects. Therefore, it is important to consider the statistical power of the study in relation to the expected effect size. As illustrated in the article by Sullivan and Feinn [8], in a study comparing two group means, the *p*-value was greater than .05, yet the effect size was 0.5. representing a medium effect. These results would be interpreted differently in this study with a sample size that was too small and not adequately powered to reach statistical significance, compared with another study with a large sample size and adequate power. Reporting the CI can help with this limitation of *p*-values, since its width partially reflects the study’s power to detect the difference.

### Be wary about multiple comparisons

If conducting multiple statistical tests or comparisons, be mindful of the increased probability of false positives (i.e., type II error). As additional statistical tests are performed, there is an additive risk that a difference will be seen based on chance alone. Some studies seem to capitalize on this mathematical phenomenon by making many comparisons to see what comes up positive (i.e., “statistical fishing expeditions”). Providing confidence intervals that transparently depict the spread and precision of the data helps address this concern. Correcting for multiple comparisons using appropriate methods (e.g., Bonferroni correction, false discovery rate control) can mitigate this threat somewhat, yet even by lowering the threshold for significance, corrections do not prevent the potentially false binary of what is significant and what is not. As an alternative to reporting multiple p-values (whether corrected or not), some leading journals have suggested reporting only a p-value for the primary outcome (e.g., *NEJM*) or entirely removing all p-values (e.g., *Nature*), and instead to focus on reporting estimated effects and 95% confidence intervals for all outcomes.

### Validate assumptions and perform sensitivity analyses

All statistical tests rest on a set of assumptions. Commonly used parametric tests (i.e., statistical tests used for data that are normally distributed), including t-tests, assume that the data are normally distributed. If such tests are used for data that are not normally distributed, the resulting *p*-value and other test statistics will lack validity (in such cases “non-parametric” tests should be used). Thus, researchers need to be familiar with the characteristics of their data before selecting statistical tests; for researchers without formal statistics training or knowledge, consultation with a statistically trained colleague is advised. After completing primary data analysis, consider sensitivity analysis to better understand the data. In our hypothetical study, for example, we might compare simulation and standard training in two separate groups – those with substantial peripheral venous line experience and those without. Such analysis will help contextualize *p*-values, whether they are greater or less than 0.05.

## Conclusion

Despite the ongoing debate around the use of *p*-values in scientific research, we advocate for continued reporting of *p*-values within scientific discourse. At the same time, we strongly support critical consideration and contextualization of this statistical concept using approaches described in this paper (and summarized in Table 1). Whenever *p*-values are reported, we recommend also reporting measures of central tendency (e.g., mean, median), dispersion (i.e., confidence intervals), absolute impact (i.e., effect size), and careful situation of results within the parameters of study design, sample size, and choice of statistical tests. Such thoughtful and informed design of analysis and reporting of statistical tests will promote responsible and informative use of *p*-values, and, ultimately, clearer communication about medical education scholarship.

**Table 1:** Pitfalls and Strategies to Mitigate Misuse of *P*-values.


PITFALLS	INSTEAD DO

Misinterpreting *p*-values for magnitude of difference	Consider the magnitude of the findings using effect sizes for example: Difference in means between groups for a scale or continuous measure. Standardized versions of these (e.g., standardized mean difference or Cohen’s *d*) Estimates of the beta coefficient for correlational studies (or standardized measures such as r, *R*^2^ or Eta-squared)

Overemphasis on statistical significance based on <.05 threshold	Consider the practical or educational significance of the estimated effect. Consider the practical/educational significance of plausible values for the effect size that are contained in the 95% Confidence Interval

Under-emphasis on *p*-values >.05 threshold	Consider sample size and sufficiency of power and the sensitivity of the measurements for interpreting the p-values. Consider Bayesian approach to estimating the parameters to increase certainty. Consider the practical or educational significance of the estimated effect. Consider whether important practically/educationally significant effects are contained in the 95% Confidence Interval

Dichotomization of *p*-values to statistically significant vs. not significant	State the null hypothesis and the justifications for the hypothesis. Use the range of plausible effect sizes contained in the 95% Confidence Interval to understand the uncertainty in the findings.

